# A Rare Case of Complete-Immunotherapy-Responsive Metastatic Non-Small Cell Lung Cancer with Long Lasting Progression-Free Survival: A Case Report

**DOI:** 10.3390/curroncol31020053

**Published:** 2024-01-26

**Authors:** Claudia De Intinis, Paolo Izzo, Massimo Codacci-Pisanelli, Luciano Izzo, Daniela Messineo, Simone Sibio, Monica Campagnol, Silvia Lai, Marcello Molle, Sara Izzo

**Affiliations:** 1 Department of Surgery “Pietro Valdoni”, Policlinico “Umberto I”, “Sapienza” University of Rome, Viale del Policlinico 155, 00161 Rome, Italy; deintinis.1891513@studenti.uniroma1.it (C.D.I.); p_izzo@hotmail.it (P.I.); massimo.codacci@uniroma1.it (M.C.-P.); simone.sibio@uniroma1.it (S.S.); m.campagnol@policlinicoumberto1.it (M.C.); 2 Department of Radiological Sciences, Oncology and Anatomo-Pathological Science, “Sapienza” University of Rome, Viale dell’Universitá 31/33, 00161 Rome, Italy; daniela.messineo@uniroma1.it; 3 Department of Translational and Precision Medicine, Nephrology Unit, “Sapienza” University of Rome, Viale dell’Universitá 31/33, 00161 Rome, Italy; silvia.lai@uniroma1.it; 4 Multidisciplinary Department of Medical-Surgical and Dental Specialties, Plastic Surgery Unit, Università degli Studi della Campania “Luigi Vanvitelli”, Piazza Luigi Miraglia 1, 80138 Naples, Italy; marcello.molle@unicampania.it (M.M.); sa_izzo@hotmail.it (S.I.)

**Keywords:** NSCLC, metastasis, immunotherapy, complete response, case report, literature review

## Abstract

Background and introduction: Lung cancer is a prevalent and deadly disease globally. Non-small cell lung cancer (NSCLC) is the most common subtype, comprising 85% of cases. Case report: A 65-year-old male ex-smoker presented to our facility with a nocturnal cough. Various investigations revealed that he had metastatic NSCLC, for which he underwent chemotherapy with cisplatin and gemcitabine, followed by immunotherapy with Nivolumab. He achieved a complete response to the therapy and has remained free from recurrence for over 7 years since the initial diagnosis. Discussion and Conclusions: The treatment of metastatic NSCLC remains a significant therapeutic challenge, but the implementation of new therapeutic techniques has expanded the possibilities of achieving complete and durable eradication of the disease.

## 1. Introduction

Lung cancer is the second most prevalent neoplastic disease globally, with 2,206,771 new cases in 2020 (11.4% of all new cancer diagnoses), and is the leading cause of cancer-related deaths, with 1,796,144 deaths in 2020 (18% of all cancer deaths) [1]. It predominantly affects males [2], and tobacco smoking is the primary etiological factor [3], with the incidence correlated with smoking prevalence in different countries [4]. Other contributing factors include exposure to radon [5], a history of chronic obstructive pulmonary disease (COPD), interstitial lung diseases [6], and the consumption of red meat [7]. Non-small cell lung cancer (NSCLC), comprising adenocarcinoma, squamous cell carcinoma, and large cell carcinoma, is the most common histological subtype, constituting 85% of cases [8]. NSCLC is often diagnosed at the onset of more advanced symptoms such as the presence of hemoptysis, chest pain, and dyspnea [9].

This study presents a case of metastatic NSCLC achieving complete remission (CR) and extremely long progression-free survival (PFS) through immunotherapy.

## 2. Case Report

**Figure 1:**
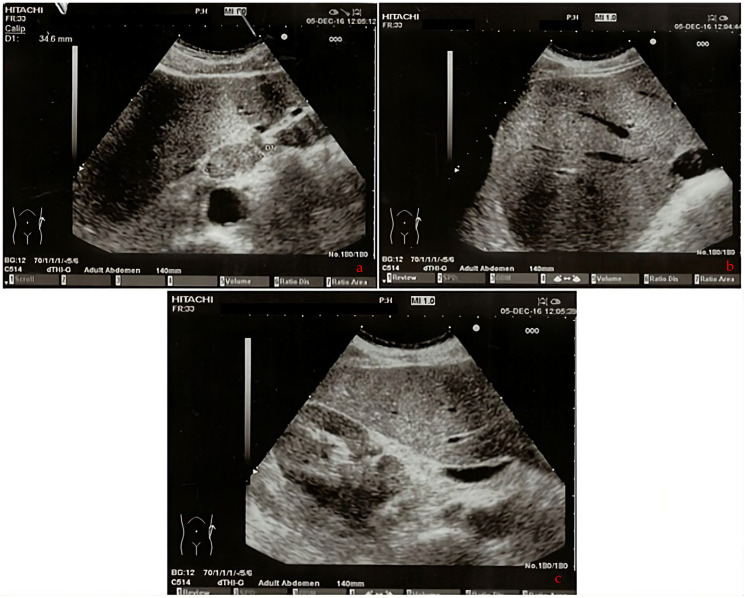
(**a**–**c**) The abdomen ultrasound images of perihepatic lymphadenopathy (2016).

A 63-year-old male patient presented at our hospital in 2016 with a worsening non-productive night cough but no dyspnea. He had a history of hypertension and a recent diagnosis of gastroesophageal reflux. A former smoker who quit 10 years ago, he had not experienced weight loss in recent months. An abdominal ultrasound revealed an area near the hepatic hilum with lobulated contours, roughly oval, displaying an iso-hypoechogenic echo structure, with a maximum diameter of 43 mm, indicating the presence of malignant lymph nodes (Figure 1a–c).

**Figure 2:**
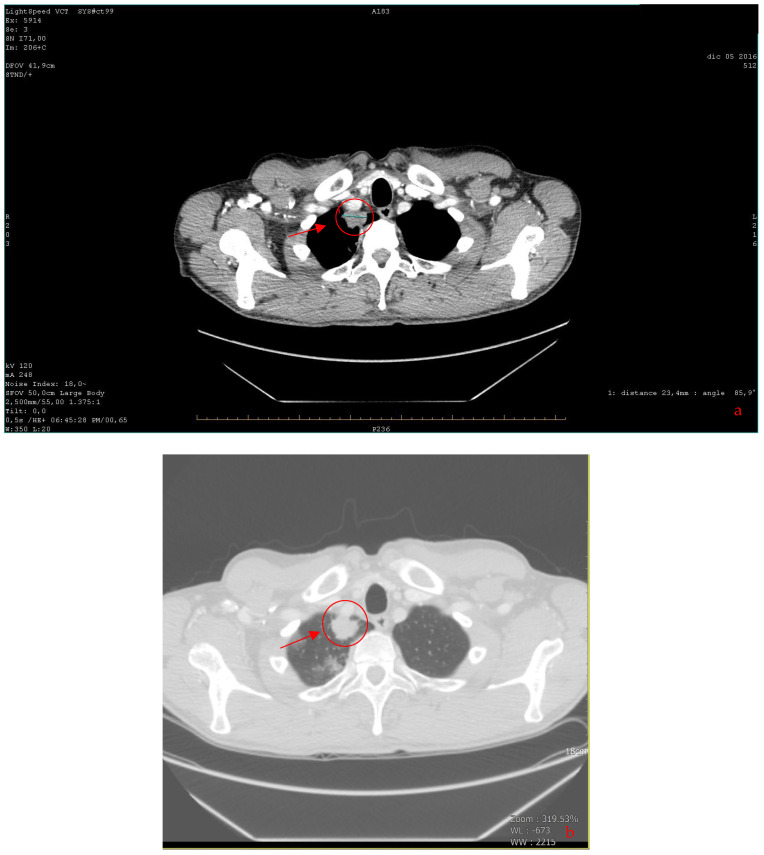
(**a**,**b**) The axial CT scan revealed a solid, nodular-like, expansive process in the right upper lobe of the lung (marked with a red circle) (**a**). To the right: lung windowing (**b**) (2016).

A total-body CT (computed tomography) scan was performed, which confirmed this suspicion and revealed the presence of a solid, nodular-like, expansive process in the right upper lobe of the lung (21 × 29 mm, adjacent to the subclavian artery) and multiple lymphadenopathies in the mediastinum (paratracheal and subcarinal) and abdomen (near the origin of the celiac trunk and periaortic lymph nodes) (Figure 2).

**Figure 3:**
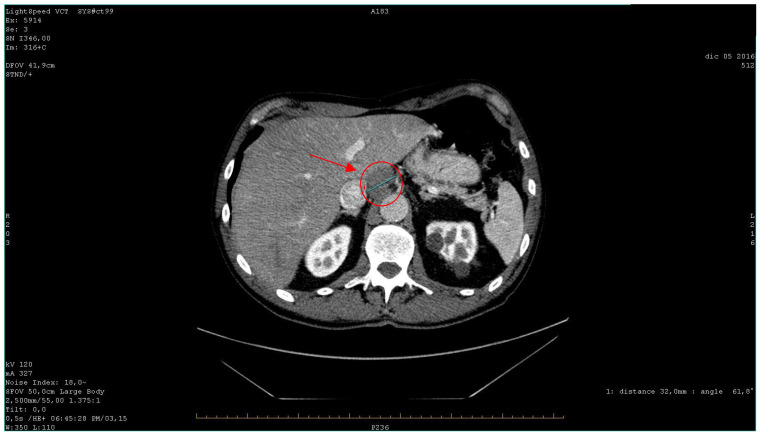
The axial CT scan of abdomen with contrast medium shows tripod lymphadenopathy (marked with a red circle) (2016).

Bronchoscopy and cytology of bronchial lavage returned negative results, while FDG PET (fluorodeoxyglucose positron emission tomography) confirmed the presence of multiple hypercapturing lesions consistent with the CT findings. Subsequently, an ultrasound-guided biopsy was performed on lymph nodes near the celiac tripod (Figure 3).

A histological examination described a poorly differentiated neoplasm suggestive of primary lung cancer. Immunohistochemistry results were positive for CKAE1/AE3, CK7, EMA, and focally for TTF1 and p63, while negative for CK5/6, CD56, Chromogranin A, and Synaptophysin. The Ki67 value was 50%. The study on EGFR for the mutation of exons 18, 19, 20, and 21 gave a negative result.

Various tumor markers were measured, indicating a primary non-small cell lung carcinoma (NSCLC): CEA was 1021.2 ng/mL (<6), Ca 19.9 was 9.0 UI/mL (<37), and NSE was 6.6 UI/mL (<10).

Given the metastatic stage of the disease, surgical removal of the primary lesion was not pursued. The patient started a chemotherapy regimen consisting of cisplatin (70 mg/m^2^ on day 1 every 21 days) and gemcitabine (1000 mg/m^2^ on days 1 and 8 every 21 days) [10,11]. After three cycles, blood tests showed hyponatremia (Na^+^ 127 mEq/mL, successfully treated) and a favorable response in cancer markers: CEA decreased from 1021.2 ng/mL to 116.2 ng/mL, and Ca15.3 decreased to 27.5 UI/mL (<30 UI/mL). Subsequent CT scans revealed a partial response to therapy with a reduction in the size of the lung tumor (from 21 × 29 mm to 10 × 15 mm) as well as thoracic and abdominal lymphadenopathies. Three additional cycles were planned, but the patient could only complete two cycles due to bone marrow suppression and subsequent anemia, which required two blood transfusions. The patient underwent bone scintigraphy, which yielded negative results for metastases in the skeletal system. Following another CT scan, which showed progression of the disease in the subdiaphragmatic lymph nodes, the patient started immunotherapy with Nivolumab (3 mg/kg every two weeks) (PD-L1 expression < 10%).

**Figure 4:**
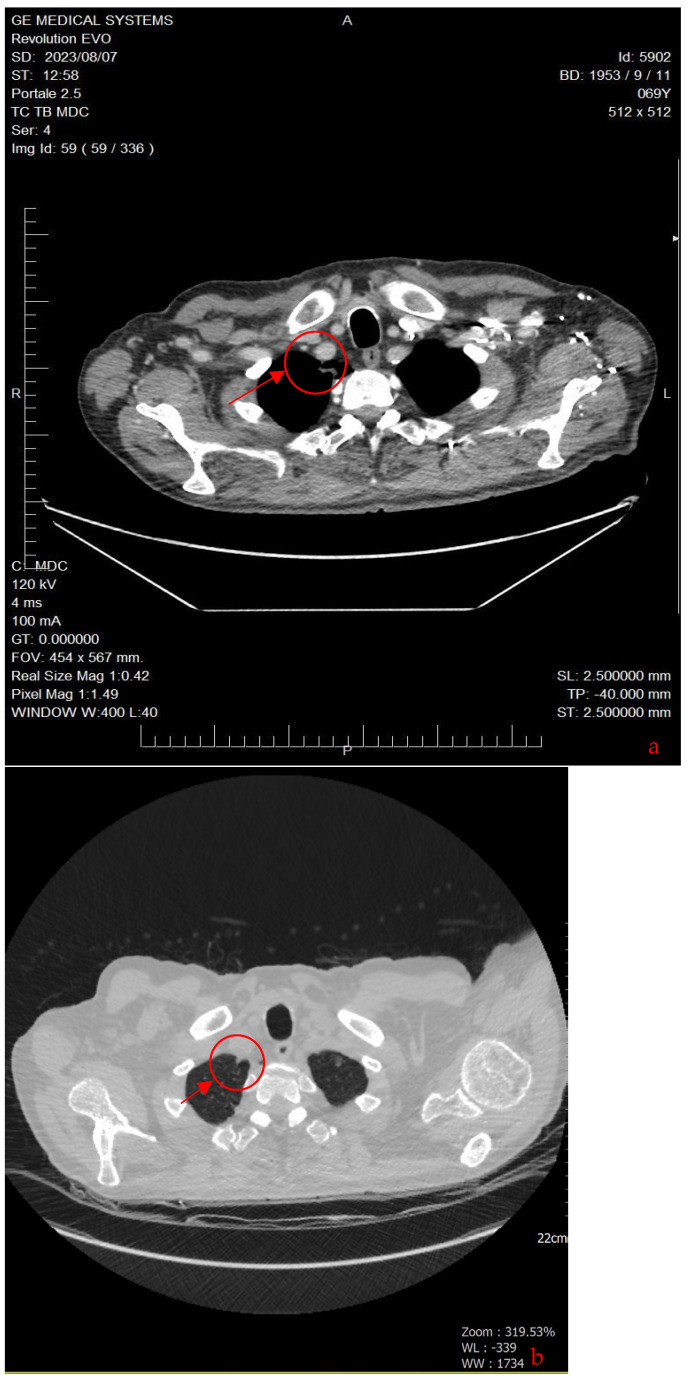
The axial CT scan shows that the solid, nodular-type, expansive process in the right upper lobe of the lung is no longer visible (red circle). This means that the nodule has disappeared or shrunk to a size that is no longer detectable on the CT scan (**a**). To the right: lung windowing (**b**) (2023).

This treatment showed a positive response with a progressive decrease in CEA levels (reaching 3 ng/mL after six months) and Ca 15.3 (22, ng/mL) and a reduction in the size of the lesions observed in subsequent CT scans (no new lymphadenopathies and a reduction in existing ones). After 18 months, Nivolumab was discontinued, as agreed with the patient, considering his positive response to therapy and the elevated risk of potential adverse reactions, compounded by his related apprehension. The patient developed pruritus unresponsive to antihistamines, which was managed with glucocorticoids. Over the next 3 years, regular monitoring with CEA, Ca15.3, and CT-PET scans indicated a complete response. CEA levels remained stable below 6 ng/mL, and subsequent CT-PET scans revealed the absence of areas exhibiting FDG uptake in the thoracic and abdominal regions. In the latest total body CT scan performed in 2023, no evidence of neoplastic disease was detected in the lung parenchyma or in the subdiaphragmatic, paratracheal, subcarinal, or periceliac lymph nodes (Figure 4). The complete timeline of the case is reported in Table 1.

## 3. Discussion and Conclusions

The patient in our case study presented with nonspecific symptoms, a history of heavy smoking (although currently not a smoker), and no previous access to cancer screening programs. The diagnosis of cancer was therefore incidental, although already at an advanced stage (T1c-N3-M1c Stage IV B according to the eighth edition of the AJCC TNM classification [12]).

It is quite common for many patients with NSCLC to be diagnosed with symptomatic disease or even at the metastatic stage (37.9%) [13]. However, this poses a problem because several studies have shown that in patients with a heavy smoking history, participation in screening programs (typically performed using low-dose CT techniques [14]) significantly reduces mortality from this condition through early detection [15,16]. Despite this, only about 4% of high-risk patients (people with a ≥20 pack-year smoking history, those who currently smoke, those who have quit within the past 15 years, and those who are between 50 and 80 years old [17]) participate in screening programs [18].

During the case management, we found that CEA (carcinoembryonic antigen) was a reliable predictor of disease progression and regression, consistent with other studies [19].

Given the absence of suitable mutations for targeted therapy, the initial line of treatment was chemotherapy with cisplatin and gemcitabine, as recommended by guidelines at the time [11]. However, this treatment regimen resulted in toxicity, including bone marrow depression, leading to the need for two blood transfusions, as well as severe hyponatremia. Despite an initial partial response, the disease progressed after treatment suspension due to the mentioned side effects.

Subsequently, the patient started immunotherapy with Nivolumab at a dose of 3 mg/kg every 3 weeks. This led to a complete response, and the patient has remained disease-free since the treatment was discontinued in mid-2018, which is now five years ago. Over time, immunotherapy has demonstrated good efficacy in the treatment of metastatic NSCLC, as supported by another works [20]. A potential concern in patient management may arise following the cessation of immunotherapy. However, our observations did not indicate any relapse or recurrence of the disease. This aligns with subsequent studies that demonstrated no declines in progression-free survival (PFS) or overall survival (OS) among oncological patients who had to discontinue Nivolumab-based immunotherapy due to adverse effects [21,22].

An apical resection of the right upper lobe was not executed for two primary reasons: Firstly, the patient was diagnosed with Stage IV disease, making surgery inconsistent with the guidelines applicable during the treatment period. Secondly, the patient declined surgical intervention [11].

It is important to state that cases like this, with patients experiencing a complete response to treatment and maintaining a disease-free state for over 5 years, are still not common in the context of NSCLC, and only anecdotally reported in the literature. Despite ongoing advancements in research and the increasingly detailed molecular characterization, tumor-related mortality and the rates of overall survival (OS), recurrence-free survival (RFS), and progression-free survival remain suboptimal [23].

Indeed, achieving such extended overall survival (OS) times (at the time of writing the article, 75 months, calculated from the date of diagnosis of the disease) in a patient with Stage IVB non-small cell lung cancer (NSCLC) and negative for EGFR/ALK mutations who could not undergo radical tumor resection is exceedingly rare. A retrospective Austrian study conducted in 2015 [13], which examined 2993 patients treated between 1989 and 2009, reported a median OS of 7.2 months for patients treated prior to 2000 and 8.4 months for patients treated after 2000, considering recurrence-free survival (RFS) for patients receiving chemotherapy (up to five different therapeutic regimens) in this patient cohort. A recent American article that considered various treatment options with chemotherapy (carboplatin in combination with paclitaxel or pemetrexed) reported similar overall survival (OS) data, with a median OS of 6.8 months for patients treated with first-line chemotherapy [24].

Higher values have been achieved with the use of immunotherapy. In the KEYNOTE-024 trial, the use of Pembrolizumab was evaluated in patients with Stage IV NSCLC and a PD-L1 tumor proportion score of 50% or greater, without EGFR/ALK mutations. In this patient pool, the median OS was reported to be 30 months [25,26].

Other trials have evaluated the response to immunotherapy with Pembrolizumab in combination with Carboplatin and Paclitaxel chemotherapy in patients with non-small cell squamous carcinoma, regardless of PD-L1 expression. These trials reported a median OS of 15.9 months in patients treated with the combination compared to 11.3 months in patients treated with chemotherapy alone. The hazard ratio for death was 0.64 in patients with a PD-L1 expression greater than 50%, 0.57 in patients with a PD-L1 expression between 1% and 49%, and 0.61 in patients with a PD-L1 expression less than 1% [27].

Similar results were also obtained in the KEYNOTE-042 trial. In this trial, the response to Pembrolizumab monotherapy was evaluated in patients with non-small cell lung cancer, regardless of PD-L1 expression. The trial demonstrated a comparable median overall survival (OS) of 16.7 months in patients treated with Pembrolizumab compared to 12.1 months in patients treated with standard chemotherapy [28,29].

Other studies have evaluated the combination of immunotherapy with stereotactic body radiation. In a 2020 study, the response was assessed in patients with metastatic NSCLC using either Pembrolizumab or Ipilimumab (anti-CTLA-4) combined with radiation treatment of 50 Gy at the site of the primary lesion. The median overall survival (OS) of patients treated with Ipilimumab was reported to be 10.7 months, while it was not determined during the study of patients treated with Pembrolizumab (with a survival rate of 66% at 18 months). These findings suggest that combining immunotherapy with stereotactic body radiation may provide favorable outcomes for patients with metastatic NSCLC [30].

In the literature, cases with an overall survival (OS) exceeding 36 months are rare. A Chinese case report from 2021 [31] described a 78-year-old male patient with a history of prolonged tobacco abuse (20 cigarettes per day) and tuberculosis, diagnosed with NSCLC Stage T1N0M0, who underwent surgery and radiation therapy. After one year of radiation treatment, the patient developed lung, liver, cardiac, and bone metastases. Subsequently, the patient received radiation therapy for the bone lesions and was administered pembrolizumab (200 mg every 21 days). The patient showed symptomatic improvement with a partial response (PR) as evidenced by CT scans. The authors reported a 7-year overall survival (OS) from the time of diagnosis, similar to the presented case, with the difference being that the patient developed metastases 3 years after the initial diagnosis, rather than at the time of initial diagnosis.

Another similar case was reported in a French study from 2022 [32], describing a 61-year-old female patient (with a heavy smoking history) with non-small cell lung cancer (NSCLC) and bone, liver, and adrenal gland metastases. Molecular screening with next-generation sequencing (NGS) of the primary lesion identified EGFR mutations (V843I, a mutation not associated with a treatment benefit from targeted therapy) and KRAS G13D. Subsequently, the patient received systemic treatment with Pembrolizumab (PD-L1 > 50%), resulting in a partial response in all tumor locations. After 30 months of progression-free survival (PFS), the patient experienced progression at the previous metastatic sites and underwent chemotherapy with pemetrexed and carboplatin, with a reported overall survival (OS) of 48 months.

Another Chinese study published in 2023 [33] reported a partial response duration of 45 months in a 66-year-old male patient undergoing systemic therapy with pembrolizumab (200 mg every 21 days). The management of the patient was challenging due to the presence of chronic renal insufficiency and the absence of immune-related adverse events (irAEs).

Another exceptional case of overall survival (OS) was described in a 2019 Japanese article [34], involving a 71-year-old male patient diagnosed with lung adenocarcinoma. Initially, the patient underwent chemotherapy with carboplatin and docetaxel, achieving a partial response that lasted for 8 months. However, the disease eventually progressed, leading to repeated cycles of re-challenging chemotherapy, which successfully controlled the disease for 6 years. Subsequently, the patient received treatment with erlotinib, resulting in a partial response. With a survival time exceeding 11 years from the time of diagnosis, this case is noteworthy. However, it differs from the case previously reported as it lacks a complete response to therapy.

A similar story is that of a 73-year-old American patient [35] who underwent immunotherapy with Nivolumab and achieved a survival time of approximately five years from the time of diagnosis. However, it should be noted that there was no complete response to the treatment.

Lastly, another noteworthy case is that reported in a 2020 Chinese study [36] of a patient with NSCLC and brain metastases who was treated with Temozolomide (150 mg/m^2^/d for 5 days every 28-day cycle) and achieved an overall survival (OS) of 55 months.

It still remains problematic to identify the reasons for this exceptional survival, firstly precisely because of the rarity of the case, which prevents us from having a sufficiently large population to study, and secondly because of the lack of sufficiently accurate data on specific patient characteristics.

It is evident that long-term survival for patients with metastatic NSCLC remains short despite the use of increasingly advanced medical and pharmaceutical technologies. In this scenario, the collection and study of exceptional patients and cases (and also studying the immunological characteristics of these patients) with long-term survival become increasingly crucial in the search for better treatment options for this patient population.

## Figures and Tables

**Table 1:** Timeline and relevant data.

Year	Event	Relevant Data
2016	Patient presentation Diagnosis Abdominal ultrasound Total body CT scan	Worsening non-productive night cough, no dyspnea. Metastatic lung cancer. Presence of malignant lymph nodes near the hepatic hilum. Solid nodular- like expansive process in the right upper lobe of the lung and multiple lymphadenopathies in the mediastinum and abdomen.
2016	US-guided biopsy Immunochemistry EGFR mutation study	Poorly differentiated neoplasm suggestive of primary lung cancer. Positive for CKAE1/AE3, CK7, EMA, focally for TTF1 and p63. Negative for CK5/6, CD56, Chromogranin A, and Synaptophysin. Ki67 value: 50%. Negative result for exons 18, 19, 20, and 21.
2016	Chemotherapy initiation	Cisplatin (70 mg/m^2^ dL every 21 days) and gemcitabine (1000 mg/m^2^, d 1, 8 every 21 days).
2016	Partial response to therapy	Reduction in the size of the lung tumor and lymphadenopathies.
2017	Bone scintigraphy	Negative results for skeletal metastases.
2017	Immunotherapy initiation	Nivolumab (3 mg/kg every two weeks).
2019	Nivolumab discontinuation	Complete response observed.
2023	Latest total body CT scan	No evidence of neoplastic disease detected.

## Data Availability

The data presented in this study are available on request from the corresponding author.

## References

[1] World Health Organization Lung Cancer. http://globocan.iarc.fr/Pages/fact_sheets_cancer.aspx.

[2] Bade B.C., Cruz C.S.D. (2020). Lung Cancer 2020: Epidemiology, Etiology, and Prevention. Clin. Chest Med..

[3] Office of the Surgeon General U.S. (1972). Public Health Service. The Health Consequences of Smoking.

[4] Bray F., Ferlay J., Soerjomataram I., Siegel R.L., Torre L.A., Jemal A. (2018). Global cancer statistics 2018: GLOBOCAN estimates of incidence and mortality worldwide for 36 cancers in 185 countries. CA Cancer J. Clin..

[5] Ajrouche R., Ielsch G., Cléro E., Roudier C., Gay D., Guillevic J., Laurier D., Le Tertre A. (2017). Quantitative Health Risk Assessment of Indoor Radon: A Systematic Review. Radiat. Prot. Dosim..

[6] Alberg A.J., Brock M.V., Ford J.G., Samet J.M., Spivack S.D. (2013). Epidemiology of lung cancer: Diagnosis and management of lung cancer, 3rd ed: American College of Chest Physicians evidence-based clinical practice guidelines. Chest.

[7] Gnagnarella P., Caini S., Maisonneuve P., Gandini S. (2018). Carcinogenicity of High Consumption of Meat and Lung Cancer Risk Among Non-Smokers: A Comprehensive Meta-Analysis. Nutr. Cancer.

[8] Travis W.D., Brambilla E., Nicholson A.G., Yatabe Y., Austin J.H.M., Beasley M.B., Chirieac L.R., Dacic S., Duhig E., Flieder D.B. (2015). The 2015 World Health Organization Classification of Lung Tumors: Impact of Genetic, Clinical and Radiologic Advances since the 2004 Classification. J. Thorac. Oncol..

[9] Siegel R.L., Miller K.D., Jemal A. (2018). Cancer statistics, 2018. CA Cancer J. Clin..

[10] Scagliotti G.V., Parikh P., Von Pawel J., Biesma B., Vansteenkiste J., Serwatowski C.M., Serwatowski P., Gatzemeier U., Digumarti R., Zukin M. (2008). Phase III Study Comparing Cisplatin Plus Gemcitabine with Cisplatin Plus Pemetrexed in Chemotherapy-Naive Patients with Advanced-Stage Non–Small-Cell Lung Cancer. J. Clin. Oncol..

[11] AIOM-Linee Guida Nazionali “Neoplasie del Polmone 2016. https://www.aiom.it/neoplasie-del-polmone-5.

[12] Detterbeck F.C., Boffa D.J., Kim A.W., Tanoue L.T. (2017). The Eighth Edition Lung Cancer Stage Classification. Chest.

[13] Kocher F., Hilbe W., Seeber A., Pircher A., Schmid T., Greil R., Auberger J., Nevinny-Stickel M., Sterlacci W., Tzankov A. (2015). Longitudinal analysis of 2293 NSCLC patients: A comprehensive study from the TYROL registry. Lung Cancer.

[14] Jemal A., Fedewa S.A. (2017). Lung Cancer Screening with Low-Dose Computed Tomography in the United States—2010 to 2015. JAMA Oncol..

[15] National Lung Screening Trial Research Team (2011). Reduced lung-cancer mortality with low-dose computed tomographic screening. N. Engl. J. Med..

[16] Walter J.E., Heuvelmans M.A., Yousaf-Khan U., Dorrius M.D., Thunnissen E., Schermann A., Groen H.J., van der Aalst C.M., Nackaerts K., Vliegenthart R. (2018). New Subsolid Pulmonary Nodules in Lung Cancer Screening: The NELSON Trial. J. Thorac. Oncol..

[17] Jonas D.E., Reuland D.S., Reddy S.M., Nagle M., Clark S.D., Weber R.P., Enyioha C., Malo T.L., Brenner A.T., Armstrong C. Screening for Lung Cancer with Low-Dose Computed Tomography: An Evidence Review for the U.S. Preventive Services Task Force. Rockville (MD): Agency for Healthcare Research and Quality (US); March 2021. (Evidence Synthesis, No. 198). https://www.ncbi.nlm.nih.gov/books/NBK568573/.

[18] Doria-Rose V.P., White M.C., Klabunde C.N., Nadel M.R., Richards T.B., McNeel T.S., Rodriguez J.L., Marcus P.M. (2012). Use of Lung Cancer Screening Tests in the United States: Results from the 2010 National Health Interview Survey. Cancer Epidemiol. Biomark. Prev..

[19] Dal Bello M.G., Filiberti R.A., Alama A., Orengo A.M., Mussap M., Coco S., Vanni I., Boccardo S., Rijavec E., Genova C. (2019). The role of CEA, CYFRA21-1 and NSE in monitoring tumor response to Nivolumab in advanced non-small cell lung cancer (NSCLC) patients. J. Transl. Med..

[20] Osmani L., Askin F., Gabrielson E., Li Q.K. (2018). Current WHO guidelines and the critical role of immunohistochemical markers in the subclassification of non-small cell lung carcinoma (NSCLC): Moving from targeted therapy to immunotherapy. Semin. Cancer Biol..

[21] Ishihara H., Nemoto Y., Nakamura K., Ikeda T., Tachibana H., Fukuda H., Yoshida K., Kobayashi H., Iizuka J., Shimmura H. (2021). Prognostic Impact of Early Treatment Interruption of Nivolumab Plus Ipilimumab Due to Immune-Related Adverse Events as First-Line Therapy for Metastatic Renal Cell Carcinoma: A Multi-Institution Retrospective Study. Target. Oncol..

[22] Kus T., Aktas G. (2020). Durable response after interruption of nivolumab in patients with metastatic renal cell carcinoma: Is renal toxicity a marker to predict the benefit of nivolumab?. J. Oncol. Pharm. Pract..

[23] Alexander M., Kim S.Y., Cheng H. (2020). Update 2020: Management of Non-Small Cell Lung Cancer. Lung.

[24] Simeone J.C., Nordstrom B.L., Patel K., Klein A.B. (2019). Treatment patterns and overall survival in metastatic non-small-cell lung cancer in a real-world, US setting. Futur. Oncol..

[25] Reck M., Rodriguez-Abreu D., Robinson A.G., Hui R., Csoszi T., Fulop A., Gottfried M., Peled N., Tafreshi A., Cuffe S. (2016). Pembrolizumab versus chemotherapy for PD-L1-positive non-small-cell lung cancer. N. Engl. J. Med..

[26] Reck M., Rodríguez–Abreu D., Robinson A.G., Hui R., Csőszi T., Fülöp A., Gottfried M., Peled N., Tafreshi A., Cuffe S. (2019). Updated Analysis of KEYNOTE-024: Pembrolizumab Versus Platinum-Based Chemotherapy for Advanced Non–Small-Cell Lung Cancer With PD-L1 Tumor Proportion Score of 50% or Greater. J. Clin. Oncol..

[27] Paz-Ares L., Luft A., Vicente D., Tafreshi A., Gumus M., Mazieres J., Hermes B., Cay Senler F., Csoszi T., Fulop A. (2018). Pembrolizumab plus Chemotherapy for Squamous Non-Small- Cell Lung Cancer. N. Engl. J. Med..

[28] Mok T.S.K., Wu Y.-L., Kudaba I., Kowalski D.M., Cho B.C., Turna H.Z., Castro G., Srimuninnimit V., Laktionov K.K., Bondarenko I. (2019). Pembrolizumab versus chemotherapy for previously untreated, PD-L1-expressing, locally advanced or metastatic non-small-cell lung cancer (KEYNOTE-042): A randomised, open-label, controlled, phase 3 trial. Lancet.

[29] Wu Y., Zhang L., Fan Y., Zhou J., Zhou Q., Li W., Hu C., Chen G., Zhang X., Zhou C. (2021). Randomized clinical trial of pembrolizumab vs chemotherapy for previously untreated Chinese patients with PD-L1-positive locally advanced or metastatic non–small-cell lung cancer: KEYNOTE-042 China Study. Int. J. Cancer.

[30] Chen D., Menon H., Verma V., Guo C., Ramapriyan R., Barsoumian H., Younes A., Hu Y., Wasley M., Cortez M.A. (2020). Response and outcomes after anti-CTLA4 versus anti-PD1 combined with stereotactic body radiation therapy for metastatic non-small cell lung cancer: Retrospective analysis of two single-institution prospective trials. J. Immunother. Cancer.

[31] Ni J., Yang L., Zhu H., Chu M., Zhang C., Zhao W., Yang M., Xu X., Zheng E., Jiang X. (2021). A patient with metastatic non-small cell lung cancer who received pembrolizumab monotherapy after stereotactic body radiotherapy had progression-free survival of nearly 5 years: A case report. Ann. Palliat. Med..

[32] Grati O.T., Zemoura L., Nhy C., Daniel C., Melaabi S., Callens C., Villars M.G., Bièche I., Girard N. (2022). Long response to immune checkpoint inhibitors in metastatic NSCLC despite EGFR germline mutation. A case report. Lung Cancer.

[33] Yun J.W., Kwon J., Lim T. (2023). Long-Term Response of Pembrolizumab in a Patient with Metastatic Squamous Non-Small Cell Lung Cancer on Hemodialysis: Case Report and Review of the Literature. Medicina.

[34] Matsuzaki T., Iwami E., Sasahara K., Kuroda A., Nakajima T., Terashima T. (2019). A case report of metastatic lung adenocarcinoma with long-term survival for over 11 years. Medicine.

[35] Baseri B., Samra B., Tam E., Chiu E., Leaf A. (2019). An Exceptional Responder to Nivolumab in Metastatic Non-Small-Cell Lung Cancer: A Case Report and Literature Review of Long-Term Survivors. Case Rep. Oncol. Med..

[36] Yang Y., Pu Y., Dai N., Wang D., Xu M.M. (2020). Complete response of radioresistant brain metastases from non-small cell lung cancer with temozolomide. Medicine.

